# Retraction: Multi-source harmonic estimation method for distribution networks based on variational modal decomposition

**DOI:** 10.1371/journal.pone.0360484

**Published:** 2026-10-20

**Authors:** The PLOS One Editors

The *PLOS One* Editors retract this article [1] due to concerns about the addition of a large number of citations to the References list without editorial approval. Many references are irrelevant to the article or corresponding cited statements, including the articles cited as References 12–16, 18, 20, 21, 24–30, 32, and 39–46. These issues raise concern about the validity and integrity of the article.

All authors either did not respond directly or could not be reached.
